# Adverse event reporting patterns of rituximab and obinutuzumab in B-cell non-Hodgkin lymphoma: a FAERS pharmacovigilance study

**DOI:** 10.3389/fimmu.2026.1855113

**Published:** 2026-07-16

**Authors:** Weihao Ma, Hongbo Fu, Yanqiong Zhou, Yuebin Wu

**Affiliations:** Department of Pharmacy, The Second Affiliated Hospital of Shantou University Medical College, Shantou, China

**Keywords:** adverse event reporting patterns, B-cell non-Hodgkin lymphoma, CD20, FAERS, obinutuzumab, rituximab

## Abstract

**Background:**

Rituximab and obinutuzumab share the CD20 target but are used across distinct B-cell non-Hodgkin lymphoma (B-NHL) subtypes, treatment backbones, and clinical eras. Their real-world adverse event (AE) reporting patterns remain incompletely characterized.

**Purpose:**

To characterize AE reporting patterns for rituximab and obinutuzumab in B-NHL using FAERS, evaluate signal retention across histological subtype and calendar-period strata and after COVID-associated report exclusion, and describe serious outcome reporting patterns during the common-market period.

**Methods:**

FAERS reports listing rituximab or obinutuzumab as the primary suspect drug with B-NHL indications from January 2004 to December 2024 were extracted. Disproportionality signals were evaluated using reporting odds ratios (RORs) and Bayesian Confidence Propagation Neural Network (BCPNN)-derived information components (ICs). Pooled B-NHL analyses identified candidate System Organ Class (SOC)- and Preferred Term (PT)-level reporting signals. Candidate signals were further evaluated across histological subtype and calendar-period strata and in a sensitivity analysis excluding reports containing COVID-associated PTs. Multivariable logistic regression assessed serious outcome reporting during the common-market period.

**Results:**

In pooled B-NHL analyses, both drugs showed overlapping SOC-level reporting domains, including hematologic, infection-related, respiratory, vascular, and hepatobiliary disorders. Infusion related reaction was the most uniformly retained PT-level signal for both drugs, whereas cytopenia-related hematologic signals showed broad but non-uniform retention. Infection-related, respiratory, and neoplasm-related findings required contextual interpretation according to reporting era, histological subtype, component PT heterogeneity, or survivorship background. Most pooled candidate clinical PT-level signals remained signal-positive after exclusion of COVID-associated reports. Obinutuzumab reports had higher adjusted odds of serious outcome reporting during 2014–2019 (aOR 1.59, 95% CI 1.42–1.77) but not during 2020–2024 (aOR 1.03, 95% CI 0.94–1.12), with a significant drug-by-period interaction.

**Conclusion:**

FAERS reports involving rituximab and obinutuzumab in B-NHL showed overlapping AE reporting domains, but pooled signals differed in retention across clinical and reporting contexts. Infusion related reaction was the clearest consistently retained PT-level finding, whereas cytopenia-related hematologic events formed a broad but non-uniform reporting domain. Signal-retention analyses may help prioritize pharmacovigilance hypotheses and setting-specific AE monitoring, but these reporting patterns require validation in longitudinal data sources and should not be interpreted as causal or direct comparative safety estimates.

## 1 Introduction

Non-Hodgkin lymphoma (NHL) is one of the most common cancers in the U.S., with incidence and mortality rates of approximately 18.7 and 4.8 per 100,000 individuals per year, respectively (1). It is a heterogeneous group of lymphoid malignancies that predominantly arise from B cells (2, 3). Among the most common subtypes of B-cell NHL (B-NHL), diffuse large B-cell lymphoma (DLBCL) is aggressive, whereas follicular lymphoma (FL) and chronic lymphocytic leukemia/small lymphocytic lymphoma (CLL/SLL) are typically indolent (4). Nearly all B-NHL subtypes are characterized by CD20, a B-cell-specific cell surface protein. This protein is expressed on the majority of B-NHL cells as well as normal B cells, but not on pro-B cells or plasma cells, making it an ideal target for immunotherapy against B-NHL (5).

Rituximab (RTX), a chimeric murine/human monoclonal antibody (mAb) against CD20, was approved by the U.S. Food and Drug Administration (FDA) in 1997 for relapsed or refractory (R/R) low-grade or follicular CD20-positive B-NHL and was subsequently expanded to first-line DLBCL, first-line FL and CLL treatment settings. Since its approval, RTX has revolutionized the treatment of B-NHL. As a single agent or in combination with standard chemotherapy, RTX significantly improves therapeutic outcomes for patients with the major B-NHL subtypes, and its use has now been extended to rarer variants (2, 5). Nevertheless, as with any antineoplastic agent, the efficacy of RTX is ultimately limited by the development of treatment resistance (6).

Obinutuzumab is a humanized glycoengineered type II anti-CD20 mAb designed to enhance antibody-dependent cellular cytotoxicity and direct cell death compared with type I anti-CD20 mAbs. It has been approved by the FDA for selected CLL and FL settings, including previously untreated CLL in combination with chlorambucil, previously untreated FL in combination with chemotherapy followed by obinutuzumab maintenance, and rituximab-exposed R/R FL in combination with bendamustine followed by obinutuzumab monotherapy. In randomized trials, obinutuzumab-containing regimens improved progression-free survival compared with RTX-containing regimens in selected FL and CLL settings (7, 8), whereas superiority was not observed in DLBCL (9).

Clinical evidence for RTX- and obinutuzumab-based regimens has been generated from randomized trials, meta-analyses, and real-world cohort studies across FL, CLL/SLL, and rituximab-refractory indolent NHL settings. These studies provide important regimen-specific evidence on adverse events (AEs), including infusion-related reactions, cytopenia-related hematologic toxicity, infections, and serious AEs (7, 8, 10–13). However, clinical trial and cohort data primarily address prespecified outcomes within defined treatment settings, whereas complementary pharmacovigilance analyses can characterize broader post-marketing AE reporting patterns, such as rare, delayed, non-prespecified, or reporting-context-dependent signals under routine practice conditions (14, 15).

Spontaneous-reporting databases provide complementary post-marketing evidence by capturing AE reports generated under routine clinical practice. However, prior pharmacovigilance studies involving RTX or obinutuzumab have largely examined broad all-indication datasets or selected AE categories (16–21), leaving uncertainty about how reporting patterns for these two mAbs vary within the clinical and temporal contexts of B-NHL. Because pooled FAERS signals may be shaped by treatment setting, disease context, reporting era, or pandemic-related reporting behavior, a common framework that incorporates these contexts is needed.

Accordingly, this study used FAERS data to characterize System Organ Class (SOC)- and Preferred Term (PT)-level AE reporting patterns for RTX and obinutuzumab in B-NHL. Pooled B-NHL disproportionality analyses were used to identify candidate reporting signals. Candidate signals were then evaluated using histological subtype-stratified, calendar-period-stratified, and COVID-associated report-exclusion analyses to assess signal retention. Serious outcome reporting models were used as complementary analyses to describe report-level seriousness patterns during the common-market period.

## 2 Methods

### 2.1 Data source

The publicly available version of FAERS data from the first quarter of 2004 to the fourth quarter of 2024 was used. FAERS is a spontaneous reporting system that includes report-level demographic and administrative information (DEMO), drug information (DRUG), reported adverse events (REAC), patient outcomes (OUTC), report sources (RPSR), therapy dates and duration (THER), and indications for use or diagnosis (INDI). Reported AE terms in REAC and indication terms in INDI were standardized using Medical Dictionary for Regulatory Activities (MedDRA, version 28.0).

### 2.2 Data processing

Duplicate reports were removed following the FDA recommendation to retain the most recent case version (22). Reports were included if RTX or obinutuzumab was listed as the primary suspect drug and the indication corresponded to B-NHL or a B-NHL subtype (**Supplementary Table 1**). Histological subtype was assigned at the report level using a mutually exclusive indication-based classification; reports with multiple mapped B-NHL indication terms were assigned according to the prespecified priority rule shown in **Supplementary Table 1**. PTs that did not represent clinical adverse events, including “off label use”, product issue-related terms, surgical or medical procedure terms, B-NHL diagnosis terms, and disease progression-related terms, were excluded from the PT-level results and clinical interpretation.

### 2.3 Disproportionality analysis

Disproportionality analyses were conducted within the B-NHL subset of FAERS. For each target drug, reports listing that drug as the primary suspect were compared with reports listing other drugs as the primary suspect within the B-NHL subset. Pooled B-NHL analyses were used to identify candidate SOC- and PT-level reporting signals. For each drug–AE pair, reporting odds ratio (ROR) and Bayesian Confidence Propagation Neural Network (BCPNN)-derived information component (IC) were calculated from the same two-by-two contingency table (**Tables 1**, **2**). RORs were reported as point estimates with the lower bound of their 95% confidence intervals (ROR_025_). BCPNN analyses used Bayesian shrinkage priors ( $\alpha_{1}=\beta_{1}=\gamma_{11}=1$; α=β=2), and IC_025_ was calculated as the lower bound of the 95% credible interval for IC according to the formulas provided in **Table 2** (23, 24). A candidate signal was defined only when all prespecified criteria were met: case count (a) ≥ 3, ROR_025_ > 1, and IC_025_ > 0.

**Table 1:** Two-by-two contingency table for disproportionality analysis.

Drug	Target AE	Other AEs
Target drug as the PS drug	a	b
Other drugs as the PS drug	c	d

a, number of reports containing both the target drug and the target AE; b, number of reports containing the target drug and non-target AEs; c, number of reports containing the target AE and other primary suspect drugs; d, number of reports containing other primary suspect drugs and non-target AEs. AE, adverse event; PS, primary suspect.

**Table 2:** Summary of disproportionality algorithms used for signal detection.

Algorithm	Equation	Criteria
ROR	$ROR=\frac{a/b}{\text{c}/d}=\frac{ad}{bc}$	a ≥ 3
	$95\%CI=e^{\ln\left(ROR\right)\pm 1.96\sqrt{\frac{1}{a}+\frac{1}{b}+\frac{1}{c}+\frac{1}{d}}}$	ROR_025_ > 1
BCPNN	$IC=\log_{2}\frac{aN}{\left(a+b)(a+c\right)}$	IC_025_ > 0
	$E(IC)=\log_{2}\frac{\left(a+\gamma_{11})(N+\alpha )(N+\beta \right)}{\left(N+\gamma )(a+b+\alpha 1)(a+c+\beta_{1}\right)}$	
	$V(IC)=\frac{1}{{\left(\ln2\right)}^{2}}[\frac{N-a+\gamma -\gamma_{11}}{\left(a+\gamma_{11})(1+N+\gamma \right)}+\frac{N-(a+b)+\alpha -\alpha_{1}}{\left(a+b+\alpha_{1})(1+N+\alpha \right)}+\frac{N-(a+c)+\beta -\beta_{1}}{\left(a+c+\beta_{1})(1+N+\beta \right)}]$	
	$\gamma =\gamma_{11}\frac{\left(N+\alpha )(N+\beta \right)}{\left(a+b+\alpha_{1})(a+c+\beta_{1}\right)}$	
	$IC_{025}=E(IC)-2\sqrt{V(IC)}$	

The a, b, c, and d terms are defined in **Table 1**; N=a+b+c+d. ROR and BCPNN were calculated from identical two-by-two contingency tables for each drug–AE pair. ROR_025_ represents the lower bound of the 95% confidence interval. The prior parameters were $\alpha_{1}=\beta_{1}=\gamma_{11}=1$; α=β=2. IC_025_ represents the lower bound of the 95% Bayesian credible interval

### 2.4 Signal-retention and sensitivity analyses

Candidate signals from the pooled B-NHL analysis were evaluated for retention across histological subtype strata, calendar-period strata, and a COVID-associated report-exclusion sensitivity analysis. Histology-stratified analyses used the mutually exclusive report-level histological subtype strata defined in Section 2.2. For calendar-period stratification, disproportionality analyses were repeated separately for 2014–2019 and 2020–2024 within the common-market period. To evaluate the influence of COVID-associated reporting, reports containing any COVID-19-related PTs identified using the Standardized MedDRA Query (SMQ) for COVID-19 (SMQ 20000237) were excluded, and SOC- and PT-level disproportionality metrics were recalculated. A pooled candidate signal was considered retained in a stratified or sensitivity analysis if it continued to meet all prespecified signal criteria. Full retention was defined as retention across all seven stratified and sensitivity analyses.

### 2.5 Serious outcome reporting analysis

Reports with serious outcomes were defined as reports containing at least one OUTC code for death, life-threatening event, hospitalization, disability, or congenital anomaly (DE, LT, HO, DS, or CA). For reports with multiple OUTC records, serious outcome status was assigned at the report level; a report was classified as having a serious outcome if any OUTC record contained one of the selected serious outcome codes. In the primary analysis, the reference category comprised reports without selected serious outcome codes, including reports with missing OUTC information and reports containing only other OUTC codes.

We first summarized unadjusted proportions of reports with serious outcomes by drug. We then fitted multivariable logistic regression models at the report level, with the main analysis restricted to the common-market period (2014–2024) and adjusted for age group, sex, calendar period, B-NHL histological subtype, and reporting country. To evaluate temporal modification, we additionally fitted a drug-by-period interaction model. Sensitivity analyses included a complete-case analysis excluding reports with unknown age or sex, an all-period model, a reporter-type-adjusted model, and an analysis restricted to reports with non-missing OUTC records.

## 3 Results

### 3.1 Descriptive characteristics of included reports

**Figure 1:**
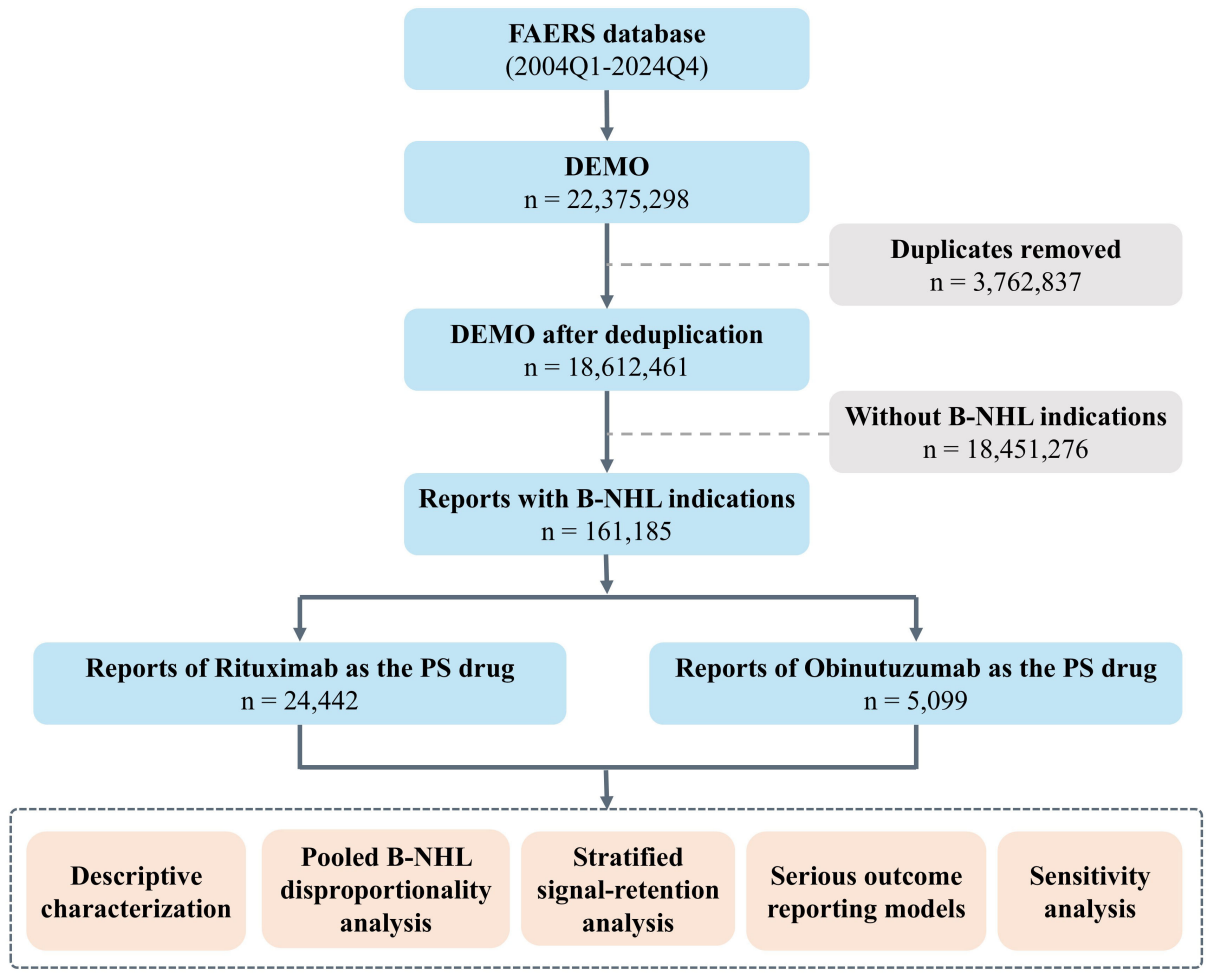
Flowchart of data screening and analysis. FAERS, FDA Adverse Event Reporting System; B-NHL, B-cell non-Hodgkin lymphoma; PS, primary suspect; Q1, first quarter; Q4, fourth quarter.

From the first quarter of 2004 to the fourth quarter of 2024, a total of 22,375,298 reports were submitted to the FAERS database. After removing duplicate reports, 18,612,461 unique reports remained. The screening process identified 24,442 reports with B-NHL indications listing RTX and 5,099 reports listing obinutuzumab as the primary suspect drug. The report selection process is shown in **Figure 1**, and descriptive report characteristics are summarized in **Table 3**.

**Table 3:** Descriptive characteristics of rituximab and obinutuzumab reports in FAERS, 2004-2024.

Characteristics	Rituximab	Obinutuzumab
Number of reports	24442	5099
Sex		
Male	10597 (43.4%)	2707 (53.1%)
Female	7258 (29.7%)	1860 (36.5%)
Unknown	6587 (26.9%)	532 (10.4%)
Age		
<18	198 (0.8%)	3 (0.1%)
18–64	6720 (27.5%)	1560 (30.6%)
>64	7549 (30.9%)	2335 (45.8%)
Unknown	9975 (40.8%)	1201 (23.6%)
Median age	65 years	68 years
Maximum age	100 years	96 years
Minimum age	4 months	9 years
Indication		
DLBCL	9266 (37.9%)	440 (8.6%)
CLL/SLL	5063 (20.7%)	2422 (47.5%)
FL	1654 (6.8%)	1562 (30.6%)
Other B-NHL	8459 (34.6%)	675 (13.2%)
Reporter country		
United States	5202 (21.3%)	1332 (26.1%)
United Kingdom	2626 (10.7%)	142 (2.8%)
France	1906 (7.8%)	301 (5.9%)
Germany	1350 (5.5%)	333 (6.5%)
China	1466 (6.0%)	476 (9.3%)
Other	11638 (47.6%)	2515 (49.3%)
Unknown	254 (1.0%)	0 (0.0%)

Values are shown as n (%) unless otherwise indicated. Percentages were calculated within each drug group. DLBCL, diffuse large B-cell lymphoma; CLL/SLL, chronic lymphocytic leukemia/small lymphocytic lymphoma; FL, follicular lymphoma; B-NHL, B-cell non-Hodgkin lymphoma.

Among reports with recorded age, the median age was 65 years (range, 4 months to 100 years) for the RTX reports and 68 years (range, 9–96 years) for the obinutuzumab reports. Male sex was reported in 43.4% of RTX reports and 53.1% of obinutuzumab reports, while sex was unspecified in 26.9% and 10.4% of reports, respectively. For indication, DLBCL was the most commonly reported indication stratum for RTX (37.9%), followed by CLL/SLL (20.7%). For obinutuzumab, CLL/SLL accounted for the largest proportion (47.5%), followed by FL (30.6%). The U.S. accounted for the largest single-country share of reports for both drugs, representing 21.3% of RTX reports and 26.1% of obinutuzumab reports.

### 3.2 Disproportionality analysis at the SOC level

**Figure 2:**
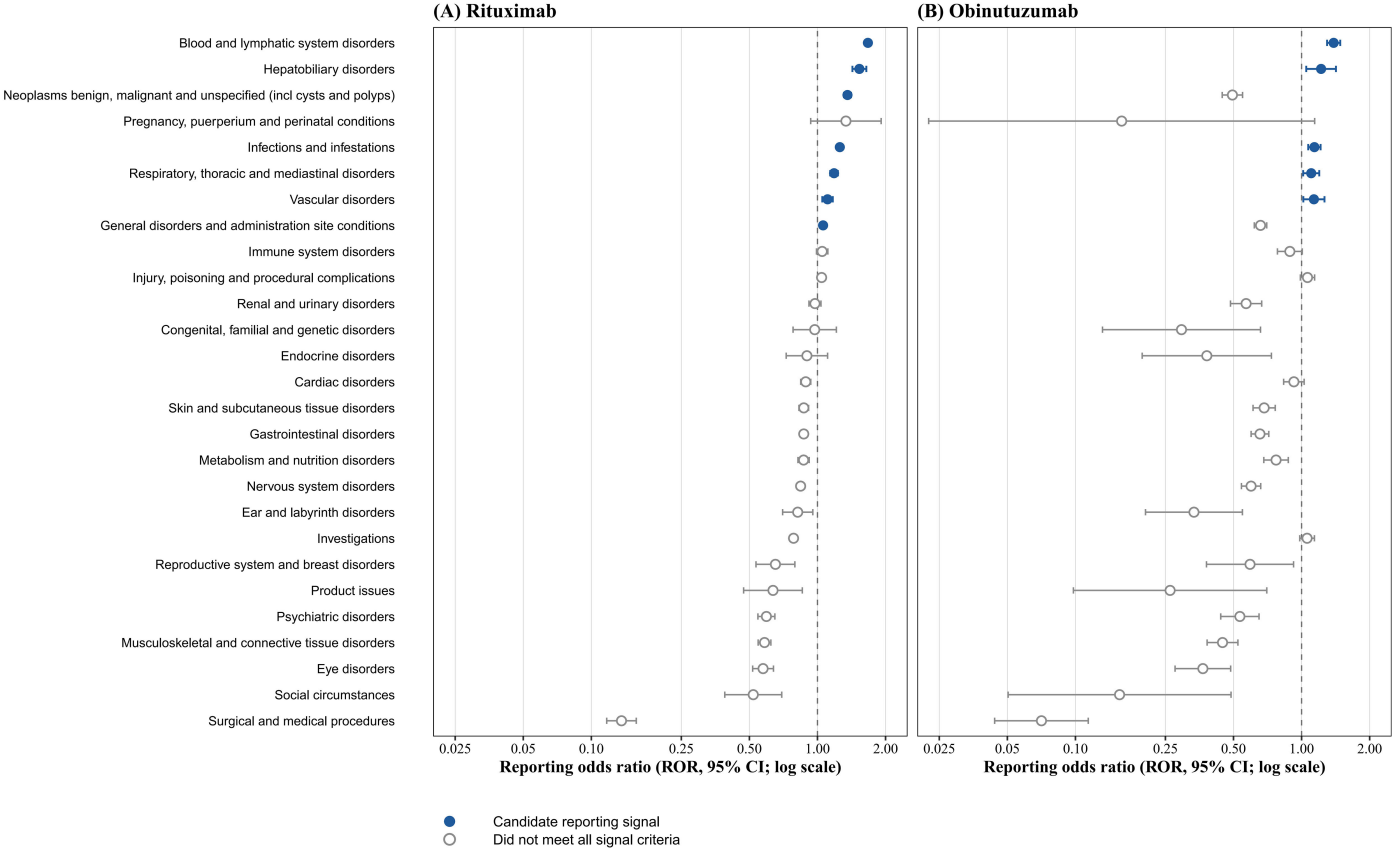
SOC-level disproportionality profiles for rituximab and obinutuzumab within B-NHL reports. Forest plots show reporting odds ratios (RORs) and 95% confidence intervals for all 27 MedDRA System Organ Classes (SOCs) in rituximab reports **(A)** and obinutuzumab reports **(B)**. Filled circles indicate SOC-level candidate signals meeting all prespecified criteria: case count **(a)** ≥ 3, ROR025 > 1, and IC025 > 0. Open circles indicate SOCs that did not meet all signal criteria. The vertical dashed line denotes ROR = 1. Both panels use the same log-scaled x-axis. SOCs are ordered by rituximab ROR.

RTX and obinutuzumab involved the same 27 SOCs (**Figure 2**). In the pooled B-NHL analysis, both drugs showed SOC-level candidate signals in Infections and infestations, Blood and lymphatic system disorders, Respiratory, thoracic and mediastinal disorders, Vascular disorders, and Hepatobiliary disorders. Two additional SOC-level signals were detected for RTX: General disorders and administration site conditions and Neoplasms benign, malignant and unspecified (incl cysts and polyps).

### 3.3 Disproportionality analysis at the PT level

At the PT level, the pooled B-NHL analysis identified overlapping candidate PT-level signals for both drugs, including neutropenia, febrile neutropenia, pyrexia, thrombocytopenia, infusion related reaction, dyspnea, chills, vomiting, hypotension, and myelosuppression (**Tables 4**, **5**). Additional prominent PT-level signals in RTX reports included pneumonia, anemia, infection, sepsis, progressive multifocal leukoencephalopathy, leukopenia, pancytopenia, and bone marrow failure. Additional prominent PT-level signals in obinutuzumab reports included platelet count decreased, neutrophil count decreased, tumor lysis syndrome, lymphocyte count decreased, oxygen saturation decreased, blood pressure decreased, chest discomfort, and flushing. The retention of these pooled candidate signals was further evaluated in stratified and sensitivity analyses.

**Table 4:** Most frequently reported PT-level candidate signals for rituximab within B-NHL reports.

PT	SOC	Number of reports	ROR (ROR_025_)	IC (IC_025_)
neutropenia	Blood and lymphatic system disorders	1695	1.83 (1.73)	0.66 (0.59)
febrile neutropenia	Blood and lymphatic system disorders	1635	1.98 (1.86)	0.75 (0.67)
pyrexia	General disorders and administration site conditions	1439	1.39 (1.31)	0.37 (0.29)
pneumonia	Infections and infestations	1226	1.13 (1.06)	0.14 (0.05)
thrombocytopenia	Blood and lymphatic system disorders	1157	1.89 (1.76)	0.71 (0.61)
anemia	Blood and lymphatic system disorders	1110	1.85 (1.72)	0.69 (0.59)
infection	Infections and infestations	896	1.94 (1.79)	0.74 (0.63)
covid-19	Infections and infestations	878	1.40 (1.30)	0.39 (0.28)
nausea	Gastrointestinal disorders	820	1.13 (1.05)	0.14 (0.03)
dyspnea	Respiratory, thoracic and mediastinal disorders	791	1.41 (1.30)	0.40 (0.28)
chills	General disorders and administration site conditions	704	2.82 (2.57)	1.12 (1.00)
infusion related reaction	Injury, poisoning and procedural complications	637	3.65 (3.30)	1.36 (1.22)
sepsis	Infections and infestations	618	1.33 (1.21)	0.33 (0.20)
vomiting	Gastrointestinal disorders	576	1.44 (1.31)	0.42 (0.29)
progressive multifocal leukoencephalopathy	Infections and infestations	550	6.02 (5.34)	1.75 (1.60)
leukopenia	Blood and lymphatic system disorders	540	2.96 (2.67)	1.17 (1.03)
pancytopenia	Blood and lymphatic system disorders	484	1.29 (1.17)	0.30 (0.16)
hypotension	Vascular disorders	477	1.31 (1.19)	0.32 (0.18)
myelosuppression	Blood and lymphatic system disorders	412	2.41 (2.15)	0.98 (0.81)
bone marrow failure	Blood and lymphatic system disorders	397	2.91 (2.57)	1.16 (0.99)

PT, Preferred Term; SOC, System Organ Class; ROR, reporting odds ratio; IC, information component. ROR_025_, lower bound of the 95% confidence interval for the ROR; IC_025_, lower bound of the 95% Bayesian credible interval for the IC. Candidate signals met all prespecified criteria: report count a ≥ 3, ROR_025_ > 1, and IC_025_ > 0. PTs are shown in descending order of report count. Complete PT-level signal tables are provided in the **Supplementary Tables**.

**Table 5:** Most frequently reported PT-level candidate signals for obinutuzumab within B-NHL reports.

PT	SOC	Number of reports	ROR (ROR_025_)	IC (IC_025_)
covid-19	Infections and infestations	427	3.47 (3.13)	1.60 (1.45)
infusion related reaction	Injury, poisoning and procedural complications	391	10.35 (9.21)	2.89 (2.73)
pyrexia	General disorders and administration site conditions	331	1.48 (1.32)	0.51 (0.34)
neutropenia	Blood and lymphatic system disorders	296	1.36 (1.21)	0.41 (0.23)
myelosuppression	Blood and lymphatic system disorders	246	6.94 (6.03)	2.47 (2.27)
febrile neutropenia	Blood and lymphatic system disorders	237	1.18 (1.03)	0.22 (0.03)
chills	General disorders and administration site conditions	223	3.70 (3.21)	1.71 (1.51)
dyspnea	Respiratory, thoracic and mediastinal disorders	206	1.71 (1.48)	0.71 (0.50)
thrombocytopenia	Blood and lymphatic system disorders	203	1.41 (1.22)	0.46 (0.25)
platelet count decreased	Investigations	175	1.44 (1.23)	0.48 (0.26)
hypotension	Vascular disorders	154	2.03 (1.72)	0.94 (0.70)
covid-19 pneumonia	Infections and infestations	124	4.38 (3.62)	1.92 (1.65)
neutrophil count decreased	Investigations	119	1.83 (1.52)	0.81 (0.54)
vomiting	Gastrointestinal disorders	114	1.29 (1.07)	0.34 (0.07)
tumor lysis syndrome	Metabolism and nutrition disorders	111	2.07 (1.70)	0.97 (0.69)
chest discomfort	General disorders and administration site conditions	96	6.11 (4.90)	2.31 (1.99)
lymphocyte count decreased	Investigations	84	3.41 (2.71)	1.61 (1.28)
oxygen saturation decreased	Investigations	73	4.38 (3.42)	1.91 (1.55)
blood pressure decreased	Investigations	63	3.92 (3.01)	1.77 (1.39)
flushing	Vascular disorders	62	7.21 (5.46)	2.47 (2.07)

PT, Preferred Term; SOC, System Organ Class; ROR, reporting odds ratio; IC, information component. ROR_025_, lower bound of the 95% confidence interval for the ROR; IC_025_, lower bound of the 95% Bayesian credible interval for the IC. Candidate signals met all prespecified criteria: report count a ≥ 3, ROR_025_ > 1, and IC_025_ > 0. PTs are shown in descending order of report count. Complete PT-level signal tables are provided in the **Supplementary Tables**.

### 3.4 Signal-retention patterns across stratified and sensitivity analyses

Candidate signals identified in the pooled B-NHL analysis were further evaluated across histological subtype strata, calendar-period strata, and the report-level COVID-associated report-exclusion sensitivity analysis.

At the SOC level, among the seven RTX and five obinutuzumab signal-positive SOCs identified in the pooled analysis, two RTX SOCs and no obinutuzumab SOCs were retained across all evaluated conditions. The fully retained RTX SOCs were Blood and lymphatic system disorders and Respiratory, thoracic and mediastinal disorders. Blood and lymphatic system disorders was the only SOC retained across both calendar periods for both drugs, although it was not retained in the obinutuzumab Other B-NHL stratum. Other RTX SOC-level signals showed mixed retention patterns: Hepatobiliary disorders and Neoplasms benign, malignant and unspecified were retained across both calendar periods but not uniformly across histological subtype strata, whereas the Infections and infestations SOC was retained in 2020–2024 and after report-level exclusion of COVID-associated reports but not in 2014–2019. In obinutuzumab analyses, SOC-level retention outside Blood and lymphatic system disorders was limited; Infections and infestations and Respiratory, thoracic and mediastinal disorders were mainly retained during 2020–2024, and the Infections and infestations SOC was not retained after report-level exclusion of COVID-associated reports.

At the PT level, after exclusion of non-clinical PTs, the retention analysis included 257 RTX and 72 obinutuzumab pooled candidate clinical PT-level signals. These signals showed more heterogeneous retention patterns than SOC-level domains. Infusion related reaction was retained across all prespecified stratified analyses and the COVID-associated report-exclusion sensitivity analysis for both drugs. Several cytopenia-related hematologic PTs also showed broad, although not uniform, retention across the evaluated analyses. Only a small subset of pooled candidate clinical PT-level signals was retained across all seven analyses, including six of 257 RTX signals and two of 72 obinutuzumab signals. Infection-related, respiratory, and COVID-associated PTs showed mixed retention patterns, with some retained across multiple analyses and others limited to selected histological subtype strata or calendar periods. After report-level exclusion of COVID-associated reports, 242 of 257 RTX and 66 of 72 obinutuzumab pooled candidate clinical PT-level signals remained signal-positive.

Consistent with the mixed PT-level retention patterns, tumor lysis syndrome (TLS) showed a notable change after histological subtype stratification. TLS was signal-positive for obinutuzumab in the pooled B-NHL analysis. Within the CLL/SLL stratum, TLS was signal-positive for both RTX and obinutuzumab, whereas the obinutuzumab TLS signal was not retained across the other histological subtype strata. The retention findings are summarized in **Table 6**, with complete SOC- and PT-level retention matrices provided in the **Supplementary Dataset**.

**Table 6:** Summary of pooled candidate signal retention across stratified and sensitivity analyses.

Retention metric	Rituximab	Obinutuzumab
Pooled signal-positive SOCs	7	5
SOCs retained across all seven analyses	2	0
SOCs retained after COVID-associated report exclusion	7/7	4/5
Pooled candidate PT-level signals	257	72
PT signals retained across all seven analyses	6	2
PT signals retained after COVID exclusion	242/257	66/72

Retention was defined as continued fulfillment of all prespecified signal criteria: report count a ≥ 3, ROR_025_ > 1, and IC_025_ > 0. The seven analyses comprised four histological subtype strata, two calendar-period strata, and the COVID-associated report-exclusion sensitivity analysis. PT-level denominators represent pooled candidate clinical PT-level signals after exclusion of non-clinical PTs from the main PT-level results. Complete SOC- and PT-level retention matrices, including retained, not retained, and not evaluable classifications, are provided in **Supplementary Dataset**.

### 3.5 Serious outcome reporting patterns

In the all-period dataset, at least one serious outcome was recorded in 12,655 RTX reports (51.8%) and 2,581 obinutuzumab reports (50.6%). Outcome information was absent from 2,735 RTX reports (11.2%) and 1,073 obinutuzumab reports (21.0%). In the common-market-period analysis (2014–2024), multivariable logistic regression adjusted for age group, sex, calendar period, B-NHL histological subtype, and reporting country showed modestly higher serious outcome reporting odds for obinutuzumab than for RTX (aOR 1.21, 95% CI 1.13–1.31; **Table 7**). This association was concentrated in the 2014–2019 period (aOR 1.59, 95% CI 1.42–1.77), was not statistically significant in 2020–2024 (aOR 1.03, 95% CI 0.94–1.12, *p* = 0.576), and was accompanied by a significant drug-by-period interaction (interaction aOR 0.65, 95% CI 0.56–0.74). Sensitivity analyses extending to the full 2004–2024 period (aOR 1.18, 95% CI 1.10–1.27) and additionally adjusting for reporter type (aOR 1.18, 95% CI 1.10–1.27) yielded estimates similar to the main model. Estimates were higher in analyses addressing missing covariate or outcome information, including the complete-case analysis excluding reports with unknown age or sex (aOR 1.48, 95% CI 1.36–1.62) and the analysis restricted to reports with non-missing OUTC records (aOR 1.41, 95% CI 1.30–1.53).

**Table 7:** Multivariable logistic regression models for serious outcome reporting during the common-market period and sensitivity analyses.

Model	Analysis population/specification	N	Estimate	aOR	95% CI	*p* value
Main model	Common-market period, adjusted model	24,420	Obinutuzumab vs Rituximab	1.21	1.13–1.31	<0.001
Drug-by-period model	Common-market period with drug-by-period interaction	24,420				
2014–2019	Period-specific drug contrast	–	Obinutuzumab vs Rituximab	1.59	1.42–1.77	<0.001
2020–2024	Period-specific drug contrast	–	Obinutuzumab vs Rituximab	1.03	0.94–1.12	0.576
Interaction	Drug-by-period interaction	–	drug-by-period	0.65	0.56–0.74	<0.001
Complete-case sensitivity	Excluding reports with unknown age or sex	14,365	Obinutuzumab vs Rituximab	1.48	1.36–1.62	<0.001
All-period sensitivity	Full 2004–2024 dataset	29,541	Obinutuzumab vs Rituximab	1.18	1.10–1.27	<0.001
Reporter-type-adjusted sensitivity	Main model additionally adjusted for reporter type	24,420	Obinutuzumab vs Rituximab	1.18	1.10–1.27	<0.001
Non-missing outcome-information sensitivity	Restricted to reports with non-missing OUTC records	20,643	Obinutuzumab vs Rituximab	1.41	1.30–1.53	<0.001

Serious outcome reporting was modeled at the report level. Unless otherwise specified, aORs compare obinutuzumab with rituximab, with rituximab as the reference category. The main model was restricted to the common-market period (2014–2024) and adjusted for age group, sex, calendar period, B-NHL histological subtype, and reporting country. Period-specific estimates were derived from the drug-by-period interaction model and share the same model population. The reporter-type-adjusted model additionally included reporter type. The all-period sensitivity model included reports from 2004–2024. The interaction estimate represents the drug-by-period interaction term. OUTC, patient outcome code; aOR, adjusted odds ratio; CI, confidence interval.

## 4 Discussion

This analysis mapped FAERS AE reporting patterns for RTX and obinutuzumab in B-NHL and showed that pooled signals differed in their retention behavior after stratified and sensitivity analyses. Retention analyses helped distinguish uniformly retained PT-level signals from broader reporting domains and signals whose interpretation changed across histological subtype, calendar period, or sensitivity analyses. In this descriptive framework, infusion related reaction was the clearest uniformly retained PT-level finding, cytopenia-related hematologic events showed broad but non-uniform retention, and infection-related, respiratory, and histological subtype-specific signals showed greater context dependence. In parallel, serious outcome reporting showed calendar-period dependence, further underscoring the importance of reporting-era context.

Among recurrent reporting patterns, infusion related reaction was the most uniformly retained PT-level finding and was retained across all evaluated analyses for both drugs. By comparison, cytopenia-related hematologic PTs showed broader domain-level consistency rather than uniform PT-level retention, with multiple cytopenia-related PTs retained across several evaluated analyses and Blood and lymphatic system disorders retained across both calendar periods for both drugs at the SOC level. The hematologic pattern is clinically coherent in B-NHL populations treated with anti-CD20-containing regimens, in which cytopenias are commonly observed and may be influenced by treatment backbone (8, 25, 26). Marrow involvement, baseline hematologic abnormalities, prior therapy, disease burden, and concomitant treatment may also contribute substantially to cytopenia reporting (27–29).

Infection-related and respiratory signals represented frequent but context-dependent reporting domains, showing greater sensitivity to calendar period and COVID-associated reporting context than infusion related reaction or the hematologic reporting domain. Exclusion of COVID-associated reports provided a contextual sensitivity check: most pooled candidate clinical PT-level signals remained signal-positive, indicating that the overall PT-level profile was not primarily driven by reports containing COVID-associated PTs. At the same time, infection-related SOC patterns remained sensitive to calendar-period stratification and the COVID-associated report-exclusion sensitivity analysis, supporting their interpretation as reporting domains that require reporting-era context. TLS further illustrated how histological subtype stratification refined pooled signal interpretation. Although TLS was signal-positive for obinutuzumab in the pooled B-NHL analysis, it was signal-positive for both RTX and obinutuzumab within the CLL/SLL stratum, whereas the obinutuzumab TLS signal was not retained across other histological strata. This pattern is more consistent with a CLL/SLL treatment-context reporting signal than with a uniform obinutuzumab-specific signal, consistent with prior pharmacovigilance observations in CLL (18). Clinically, TLS risk in CLL/SLL can be modified by tumor burden, renal vulnerability, monitoring intensity, and treatment backbone (30, 31).

Separately, Neoplasms benign, malignant and unspecified (incl cysts and polyps) emerged as a broad SOC-level reporting domain in RTX reports. In stratified analyses, this neoplasm-related SOC was retained in most evaluated RTX analyses, suggesting that the pooled SOC-level signal was also observed across several analytic contexts. The component PTs within this SOC were heterogeneous, including myelodysplastic syndrome, acute myeloid leukemia, squamous cell carcinoma, breast cancer, and lung adenocarcinoma, among other hematologic or solid-organ neoplasm-related terms, indicating that the signal did not correspond to a single malignancy outcome. This pattern should be interpreted against the background of NHL survivorship, in which second primary malignancies and therapy-related myeloid neoplasms are recognized concerns and may vary by lymphoma subtype, treatment era, and length of follow-up (32, 33). The longer post-marketing history and accumulated reporting window for RTX may have contributed to this reporting pattern, although FAERS cannot determine the relative contribution of this factor. Available randomized and pooled clinical evidence has not established RTX itself as an independent driver of second primary malignancy (34). Nevertheless, the FAERS finding identifies a heterogeneous neoplasm-related reporting domain in RTX reports that warrants further evaluation in registries, longitudinal real-world datasets, and extended clinical trial follow-up.

Beyond AE signal retention, calendar period also shaped report-level seriousness patterns. In the serious outcome reporting model, higher odds of serious outcome reporting for obinutuzumab were concentrated in 2014–2019 and attenuated toward the null in 2020–2024. This time-dependent pattern was directionally consistent with the descriptive decline in serious outcome proportions among obinutuzumab reports across the two calendar periods, and the adjusted estimates indicated that the 2014–2019 association persisted after adjustment for available report-level characteristics. The concentration of elevated serious outcome reporting odds during the earlier post-marketing period may be consistent with selective enrichment of serious reports rather than a stable difference across the full common-market period. Sensitivity analyses also suggested that model estimates were affected by demographic and outcome-information completeness. Together, these findings highlight the importance of accounting for reporting-era context and missingness in demographic and outcome-information fields when interpreting serious outcome reporting in FAERS.

## 5 Limitations

These findings should be interpreted within the scope of post-marketing spontaneous reporting data. FAERS does not capture exposure denominators, treatment regimens, disease severity, tumor burden, supportive-care practices, or concomitant medications; observed reporting patterns may therefore reflect treatment-backbone effects, clinical context, or disease biology rather than antibody-specific toxicity. RTX and obinutuzumab differ substantially in approval era, indication distribution, and accumulated reporting volume, limiting direct comparative safety inference. Missing demographic and outcome information may have influenced serious outcome models: 40.8% of RTX reports and 23.6% of obinutuzumab reports lacked recorded age, and 11.2% and 21.0% lacked outcome information, respectively; sensitivity analyses suggested that estimates were affected by this missingness, although the direction of association remained consistent. Stratified analyses reduced cell counts and rendered some PTs unevaluable in specific strata, particularly for obinutuzumab outside CLL/SLL and FL; absence of a retained signal in an individual stratum should therefore not be interpreted as evidence of absence of risk. Finally, exclusion of non-clinical PTs was performed at the PT level and did not represent case-level classification of on-label versus off-label treatment, because FAERS indication fields do not reliably capture full prescribing context.

## 6 Conclusion

This FAERS-based analysis characterized AE reporting patterns for RTX and obinutuzumab in B-NHL and showed that pooled disproportionality signals varied in their retention across histological subtype, calendar period, and the COVID-associated report-exclusion sensitivity analysis. Infusion related reaction was the most uniformly retained PT-level signal for both drugs, whereas cytopenia-related hematologic events formed a broad but non-uniform reporting domain. Infection-related, respiratory, TLS-related, and neoplasm-related findings required more contextual interpretation, reflecting the influence of reporting era, histological subtype, component PT heterogeneity, treatment context, or survivorship background. Serious outcome reporting also showed calendar-period dependence, highlighting the importance of reporting-era context and outcome-information completeness when interpreting report-level seriousness patterns. Overall, signal-retention analyses provided a context-aware approach for distinguishing consistently retained reporting signals from broader or more context-dependent reporting domains in spontaneous reporting data. These findings support pharmacovigilance hypothesis generation and setting-specific AE monitoring, but require validation in longitudinal data sources and should not be interpreted as causal or direct comparative safety estimates.

## Data Availability

The original contributions presented in the study are included in the article/**Supplementary Material**. Further inquiries can be directed to the corresponding author.
