# Supplementary material for: Adverse event reporting patterns of rituximab and obinutuzumab in B-cell non-Hodgkin lymphoma: a FAERS pharmacovigilance study
Source: Front Immunol. 2026 Jul 16;17:1855113. doi: 10.3389/fimmu.2026.1855113 (PMC13422546; doi:10.3389/fimmu.2026.1855113)
Supplement: Supplementary file 1 [file Table1.docx]

**Supplementary Table S1**. Operational mapping of B-NHL indication terms to histological strata.

| **Fine histology category** | **MedDRA HLT name** | **HLT code** | **Main analysis stratum** |
| --- | --- | --- | --- |
| DLBCL | Diffuse large B-cell lymphomas | 10012819 | DLBCL |
| CLL/SLL | Leukaemias chronic lymphocytic | 10024295 | CLL/SLL |
|  | B-cell small lymphocytic lymphomas | 10003909 |  |
| FL | Follicular lymphomas | 10085262 | FL |
| PMBCL | Primary mediastinal large B-cell lymphomas | 10036711 | Other B-NHL |
| MCL | Mantle cell lymphomas | 10026798 |  |
| MZL | Extranodal marginal zone B-cell lymphomas (low grade B-cell) | 10015821 |  |
|  | Nodal marginal zone B-cell lymphomas | 10029461 |  |
|  | Splenic marginal zone lymphomas | 10041650 |  |
|  | Marginal zone lymphomas NEC | 10077528 |  |
| BL/BLL | Burkitt's lymphomas | 10006596 |  |
|  | High grade B-cell lymphomas Burkitt-like lymphoma | 10020068 |  |
| LPL/WM | Lymphoplasmacytoid lymphomas/immunocytomas | 10025343 |  |
|  | Waldenstrom's macroglobulinaemias | 10047802 |  |
| B-LBL | Precursor B-lymphoblastic lymphomas | 10036524 |  |
| B-NHL NOS | B-cell lymphomas NEC | 10003900 |  |
|  | B-cell unclassifiable lymphomas | 10003921 |  |

Note: Indication PTs were mapped to MedDRA HLTs using MedDRA version 28.0. For the main subgroup analysis, reports were assigned to one of four mutually exclusive operational strata: DLBCL, CLL/SLL, FL, and Other B-NHL. Reports containing multiple mapped B-NHL indication terms were assigned according to the prespecified priority order: DLBCL, CLL/SLL, FL, and Other B-NHL.

**Supplementary Table S2**. Excluded non-clinical PTs

| **PT Code** | **PT Name** |
| --- | --- |
| 10000383 | accidental poisoning |
| 10002227 | anaplastic large cell lymphoma t- and null-cell types |
| 10002449 | angioimmunoblastic t-cell lymphoma |
| 10003010 | appendicectomy |
| 10003899 | b-cell lymphoma |
| 10003902 | b-cell lymphoma recurrent |
| 10003903 | b-cell lymphoma refractory |
| 10003908 | b-cell small lymphocytic lymphoma |
| 10003911 | b-cell small lymphocytic lymphoma recurrent |
| 10006595 | burkitt's lymphoma |
| 10006598 | burkitt's lymphoma recurrent |
| 10007953 | central nervous system lymphoma |
| 10008611 | cholecystectomy |
| 10008958 | chronic lymphocytic leukaemia |
| 10008961 | chronic lymphocytic leukaemia recurrent |
| 10010162 | complications of bone marrow transplant |
| 10010253 | concomitant disease aggravated |
| 10010264 | condition aggravated |
| 10011677 | cutaneous t-cell lymphoma |
| 10011906 | death |
| 10012818 | diffuse large b-cell lymphoma |
| 10012821 | diffuse large b-cell lymphoma recurrent |
| 10012822 | diffuse large b-cell lymphoma refractory |
| 10012826 | diffuse large b-cell lymphoma stage iv |
| 10013709 | drug ineffective |
| 10013710 | drug interaction |
| 10015823 | extranodal marginal zone b-cell lymphoma (malt type) recurrent |
| 10015866 | extravasation |
| 10017973 | gastrointestinal lymphoma |
| 10018875 | haemodialysis |
| 10019053 | hairy cell leukaemia |
| 10020096 | hip arthroplasty |
| 10020206 | hodgkin's disease |
| 10020244 | hodgkin's disease nodular sclerosis |
| 10022062 | injection site extravasation |
| 10022519 | intensive care |
| 10024288 | leukaemia |
| 10025250 | lymphocyte adoptive therapy |
| 10025270 | lymphocytic leukaemia |
| 10025310 | lymphoma |
| 10025342 | lymphoplasmacytoid lymphoma/immunocytoma |
| 10026800 | mantle cell lymphoma recurrent |
| 10026801 | mantle cell lymphoma refractory |
| 10026805 | mantle cell lymphoma stage iv |
| 10027091 | medication error |
| 10027452 | metastases to bone |
| 10027457 | metastases to liver |
| 10027458 | metastases to lung |
| 10027459 | metastases to lymph nodes |
| 10027480 | metastatic malignant melanoma |
| 10029547 | non-hodgkin's lymphoma |
| 10029600 | non-hodgkin's lymphoma recurrent |
| 10029601 | non-hodgkin's lymphoma refractory |
| 10033295 | overdose |
| 10034623 | peripheral t-cell lymphoma unspecified |
| 10035543 | platelet transfusion |
| 10036181 | porphyria |
| 10036410 | postoperative wound infection |
| 10036556 | pregnancy |
| 10036590 | premature baby |
| 10036595 | premature delivery |
| 10036713 | primary mediastinal large b-cell lymphoma recurrent |
| 10036888 | prolymphocytic leukaemia |
| 10036909 | prostate cancer metastatic |
| 10037621 | pyloric stenosis |
| 10038111 | recurrent cancer |
| 10041652 | splenic marginal zone lymphoma recurrent |
| 10042434 | sudden death |
| 10042971 | t-cell lymphoma |
| 10043414 | therapeutic response decreased |
| 10043417 | therapeutic response unexpected |
| 10043903 | tobacco abuse |
| 10045169 | tumour flare |
| 10047715 | von willebrand's disease |
| 10047801 | waldenstrom's macroglobulinaemia |
| 10047804 | waldenstrom's macroglobulinaemia recurrent |
| 10048031 | wound dehiscence |
| 10048038 | wound infection |
| 10048919 | performance status decreased |
| 10049414 | treatment noncompliance |
| 10049418 | sudden cardiac death |
| 10049993 | cardiac death |
| 10050206 | macrophage activation |
| 10050513 | metastatic renal cell carcinoma |
| 10051077 | post procedural haemorrhage |
| 10051082 | therapy non-responder |
| 10051083 | therapy responder |
| 10051099 | catheter site haemorrhage |
| 10051118 | drug ineffective for unapproved indication |
| 10051358 | post transplant lymphoproliferative disorder |
| 10051398 | malignant neoplasm progression |
| 10051664 | metastases to abdominal wall |
| 10051676 | metastases to peritoneum |
| 10051696 | metastases to meninges |
| 10052358 | colorectal cancer metastatic |
| 10052428 | wound |
| 10053180 | leukaemia cutis |
| 10053181 | therapeutic response delayed |
| 10053377 | central venous catheterisation |
| 10053762 | off label use |
| 10054112 | hospitalisation |
| 10055114 | colon cancer metastatic |
| 10056407 | refusal of treatment by patient |
| 10057335 | therapeutic embolisation |
| 10057362 | underdose |
| 10057677 | transplant |
| 10057765 | procedural complication |
| 10058019 | cancer pain |
| 10058046 | post procedural complication |
| 10058717 | chronic lymphocytic leukaemia transformation |
| 10058728 | richter's syndrome |
| 10059282 | metastases to central nervous system |
| 10059443 | skin neoplasm excision |
| 10059866 | drug resistance |
| 10060769 | therapeutic product ineffective |
| 10060933 | adverse event |
| 10061105 | dialysis |
| 10061232 | lymphoproliferative disorder |
| 10061275 | mantle cell lymphoma |
| 10061289 | metastatic neoplasm |
| 10061309 | neoplasm progression |
| 10061355 | poisoning |
| 10061520 | ill-defined disorder |
| 10061623 | adverse drug reaction |
| 10061730 | bone marrow transplant |
| 10061818 | disease progression |
| 10061819 | disease recurrence |
| 10061850 | extranodal marginal zone b-cell lymphoma (malt type) |
| 10061858 | fluid replacement |
| 10061864 | neoplasm recurrence |
| 10062113 | splenic marginal zone lymphoma |
| 10062132 | tooth extraction |
| 10062194 | metastasis |
| 10062247 | skin graft |
| 10062355 | unevaluable event |
| 10062489 | leukaemia recurrent |
| 10063569 | metastatic squamous cell carcinoma |
| 10063581 | stem cell transplant |
| 10063916 | metastatic gastric cancer |
| 10064306 | incorrect drug administration rate |
| 10064344 | lymphoma transformation |
| 10064355 | incorrect dose administered |
| 10064385 | circumstance or information capable of leading to medication error |
| 10064571 | gene mutation |
| 10064774 | infusion site extravasation |
| 10065154 | therapy cessation |
| 10066152 | transfusion |
| 10066377 | therapy interrupted |
| 10066476 | haematological malignancy |
| 10066901 | treatment failure |
| 10067268 | post procedural infection |
| 10067450 | endotracheal intubation |
| 10067476 | apparent death |
| 10067477 | cytogenetic abnormality |
| 10067482 | no adverse event |
| 10068349 | epstein-barr virus associated lymphoproliferative disorder |
| 10069327 | product quality issue |
| 10069754 | acquired gene mutation |
| 10070863 | toxicity to various agents |
| 10071404 | foetal exposure during pregnancy |
| 10071408 | maternal exposure during pregnancy |
| 10071441 | epstein-barr virus associated lymphoma |
| 10071541 | metastatic lymphoma |
| 10073085 | prescribed underdose |
| 10073513 | exposure during pregnancy |
| 10073759 | catheter site extravasation |
| 10074903 | intentional product misuse |
| 10074941 | therapeutic response changed |
| 10075173 | bone marrow infiltration |
| 10075324 | ocular lymphoma |
| 10075648 | braf gene mutation |
| 10076308 | intentional product use issue |
| 10076309 | product use issue |
| 10076313 | remission not achieved |
| 10076476 | product use in unapproved indication |
| 10076573 | wrong technique in product usage process |
| 10076596 | marginal zone lymphoma |
| 10076869 | product preparation error |
| 10077403 | hairy cell leukaemia recurrent |
| 10077533 | marginal zone lymphoma recurrent |
| 10077534 | marginal zone lymphoma refractory |
| 10078115 | therapy partial responder |
| 10078504 | contraindicated product administered |
| 10078575 | therapeutic response shortened |
| 10078798 | oxygen therapy |
| 10079212 | intercepted product storage error |
| 10079213 | intercepted product preparation error |
| 10079221 | intentional dose omission |
| 10079317 | drug effective for unapproved indication |
| 10079523 | catheter site thrombosis |
| 10079843 | product storage error |
| 10080080 | symptom recurrence |
| 10080202 | double hit lymphoma |
| 10080215 | high-grade b-cell lymphoma |
| 10080359 | product dispensing error |
| 10081202 | incorrect route of product administration |
| 10081478 | poor quality product administered |
| 10081572 | inappropriate schedule of product administration |
| 10081594 | procedural failure |
| 10081653 | bing-neel syndrome |
| 10081743 | intercepted product prescribing error |
| 10081770 | product prescribing error |
| 10082200 | therapeutic product effect incomplete |
| 10082201 | therapeutic product effect decreased |
| 10082239 | cancer fatigue |
| 10083365 | drug effect less than expected |
| 10084221 | loss of therapeutic response |
| 10084406 | product dose omission issue |
| 10085087 | treatment delayed |
| 10085126 | follicular lymphoma stage iv |
| 10085127 | follicular lymphoma stage iii |
| 10085128 | follicular lymphoma |
| 10089556 | follicular lymphoma recurrent |
| 10090210 | congenital aplasia |

**Supplementary Methods**

**1. FAERS Data Extraction and Deduplication**

Quarterly ASCII data files from the FDA Adverse Event Reporting System (FAERS) were downloaded from the U.S. FDA FAERS public database for Q1 2004 through Q4 2024. Seven data tables were used: DEMO, DRUG, REAC, OUTC, INDI, RPSR, and THER. Duplicate reports were removed by retaining the most recent CASEVERSION for each CASEID, following the FDA-recommended deduplication procedure. Reported adverse events (AEs) and indication terms were standardized and mapped using MedDRA version 28.0.

Reports for rituximab and obinutuzumab were identified from the DRUG table by case-insensitive matching of the DRUGNAME or PROD_AI fields against prespecified generic and brand-name terms. For rituximab, the search terms included rituximab, Rituxan, MabThera, Riabni, Rituxan Hycela, Ruxience, and Truxima. For obinutuzumab, the search terms included obinutuzumab and Gazyva. The analysis was restricted to reports in which rituximab or obinutuzumab was recorded as the primary suspect drug (ROLE_COD = “PS”).

B-NHL indications were identified from the INDI table by mapping indication PTs to the MedDRA High Level Terms (HLTs) listed in Supplementary Table S1. Reports with at least one INDI record matching a B-NHL HLT were retained. Histological subtype was assigned at the report level using a mutually exclusive classification: DLBCL (HLT 10012819), FL (HLT 10085262), CLL/SLL (HLTs 10024295 and 10003909), and Other B-NHL (all remaining B-NHL HLTs). For reports with multiple mapped B-NHL indication terms, the prespecified priority rule DLBCL > CLL/SLL > FL > Other B-NHL was applied.

**2. Disproportionality Calculation**

For each drug–AE pair, a two-by-two contingency table was constructed according to the definitions provided in main text Table 1. The comparator group comprised B-NHL reports listing drugs other than the target drug as the primary suspect within the corresponding analysis set. The reporting odds ratio (ROR) and Bayesian Confidence Propagation Neural Network (BCPNN)-derived information component (IC) were calculated from the same four-cell table. ROR, ROR025, IC, and IC025 were calculated according to the formulas specified in main text Table 2. A candidate signal was defined only when all three prespecified criteria were met: case count a ≥ 3, ROR025 > 1, and IC025 > 0.

Separate analyses were performed at the System Organ Class (SOC) level and the PT level. For SOC-level analyses, REAC records were mapped to SOCs using the MedDRA hierarchy. PTs that did not represent clinical AEs, including off-label use, product issue-related terms, surgical and medical procedure terms, B-NHL diagnosis terms, and disease progression-related terms, were excluded from the PT-level analyses and clinical interpretation. This exclusion was performed at the PT level and did not represent case-level classification of on-label versus off-label treatment.

**3. Signal-Retention and Serious Outcome Analysis**

Candidate signals identified in the pooled B-NHL analysis were further evaluated in retention analyses across histological subtype strata (DLBCL, CLL/SLL, FL, and Other B-NHL), calendar-period strata (2014–2019 and 2020–2024 within the common-market period), and a COVID-associated report-exclusion sensitivity analysis. For the COVID-associated report exclusion, reports containing any COVID-19-related PT identified using the Standardised MedDRA Query for COVID-19 (SMQ 20000237) were removed, and disproportionality metrics were recalculated. A pooled candidate signal was considered retained in each analysis if it continued to meet all three prespecified signal criteria.

For analyzed pooled, stratified, and sensitivity-analysis datasets, the corresponding a, b, c, d, ROR, ROR025, IC, and IC025 values are provided in the Supplementary Dataset. Retention status (retained, not retained, or not evaluable) and candidate-signal status are indicated where applicable. Not evaluable was assigned when the case count was below the minimum threshold (a < 3) in that analysis.

Serious outcomes were defined as reports containing at least one OUTC code for death (DE), life-threatening event (LT), hospitalization (HO), disability (DS), or congenital anomaly (CA), identified from outc_code fields. Reports with multiple OUTC records were classified as serious if any record contained one of the specified codes. Reports without any selected serious outcome code were used as the reference group in the primary models, including reports with no OUTC record and reports with only other OUTC codes. Multivariable logistic regression models for serious outcome reporting were fitted at the report level. The main model was restricted to the common-market period (2014–2024) and adjusted for age group, sex, calendar period, B-NHL histological subtype, and reporting country. A drug-by-period interaction model was additionally fitted. Sensitivity analyses included a complete-case analysis excluding reports with unknown age or sex, an all-period model (2004–2024), a reporter-type-adjusted model, and an analysis restricted to reports with non-missing OUTC records.
